# Use of antibiotics and risk of type 2 diabetes, overweight and obesity: the Cardiovascular Risk in Young Finns Study and the national FINRISK study

**DOI:** 10.1186/s12902-022-01197-y

**Published:** 2022-11-18

**Authors:** Joel Nuotio, Teemu Niiranen, Tomi T. Laitinen, Jessica Miller, Matthew A. Sabin, Aki S. Havulinna, Jorma S. A. Viikari, Tapani Rönnemaa, Nina Hutri-Kähönen, Tomi P. Laitinen, Päivi Tossavainen, Veikko Salomaa, Olli T. Raitakari, David P. Burgner, Markus Juonala

**Affiliations:** 1 Research Centre of Applied and Preventive Cardiovascular Medicine, University of Turku, Kiinamyllynkatu 10, FIN-20520 Turku, Finland; 2 Centre for Population Health Research, University of Turku and Turku University Hospital, Turku, Finland; 3 Heart Center, Turku University Hospital and University of Turku, Turku, Finland; 4 Murdoch Children’s Research Institute, Melbourne, Victoria Australia; 5 Finnish Institute for Health and Welfare, Helsinki, Finland; 6 Department of Internal Medicine, University of Turku, Turku, Finland; 7 Division of Medicine, Turku University Hospital, Turku, Finland; 8 Paavo Nurmi Centre, Sports and Exercise Medicine Unit, Department of Physical Activity and Health, University of Turku, Turku, Finland; 9 Department of Paediatrics, The University of Melbourne, Melbourne, Victoria Australia; 10 Department of Endocrinology and Diabetes, The Royal Children’s Hospital, Parkville, Victoria Australia; 11 Institute for Molecular Medicine Finland, HiLIFE, University of Helsinki, Helsinki, Finland; 12 Department of Pediatrics, Tampere University and Tampere University Hospital, Tampere, Finland; 13 Department of Clinical Physiology and Nuclear Medicine, University of Eastern Finland and Kuopio University Hospital, Kuopio, Finland; 14 Department of Pediatrics, Oulu University Hospital and University of Oulu, Oulu, Finland; 15 Department of Clinical Physiology and Nuclear Medicine, Turku University Hospital, Turku, Finland

**Keywords:** Antibiotics, Type 2 diabetes, Obesity

## Abstract

**Purpose:**

To investigate whether exposure to systemic antibiotics influences the risk of developing type 2 diabetes and overweight/obesity.

**Methods:**

The study sample comprised 2209 (110 with incident diabetes) participants from the population-based Cardiovascular Risk in Young Finns Study (YFS) aged 24–39 years in 2001. The exposure was national linked register data on purchased antibiotic courses between 1993 and 2001. Clinical examinations including BMI were conducted in 2001, 2007 and 2011. Participants with prevalent diabetes in 2001 were excluded. Data on type 2 diabetes was also obtained from two national registers until 2017. Data from four population-based National FINRISK studies were used for replication (*N* = 24,674, 1866 with incident diabetes).

**Results:**

Prior antibiotic exposure (> 5 versus 0–1 antibiotic courses) was associated with subsequent type 2 diabetes in both YFS (OR 2.29; 95%CI 1.33–3.96) and FINRISK (HR 1.73; 95%CI 1.51–1.99). An increased risk for type 2 diabetes was observed in YFS (OR 1.043; 95%CI 1.013–1.074) and FINRISK (HR 1.022; 95%CI 1.016–1.029) per course. Exposure to antibiotics increased the risk of overweight/obesity (BMI > 25 kg/m^2^) after a 10-year follow-up in YFS (OR 1.043; 95%CI 1.019–1.068) and in FINRISK (OR 1.023; 95%CI 1.018–1.029) at baseline per antibiotic course. Adjustments for confounders from early life in YFS and at baseline in FINRISK, including BMI, socioeconomic status, smoking, insulin, blood pressure, and physical activity, did not appreciably alter the findings.

**Conclusion:**

Our results show that exposure to antibiotics was associated with increased risk for future type 2 diabetes and overweight/obesity and support judicious antibiotic prescribing.

**Supplementary Information:**

The online version contains supplementary material available at 10.1186/s12902-022-01197-y.

## Introduction

The global disease burden from type 2 diabetes continues to increase worldwide, largely driven by the rising prevalence of obesity [1–3]. In 2015, excess bodyweight contributed to an estimated 4.0 million deaths, and the majority were due to cardiovascular disease and type diabetes [4]. The global prevalence of type 2 diabetes has been projected to increase from 415 million (1 in 11 adults) in 2015 to 642 million (1 in 10 adults) by 2040, resulting in a substantial further increase in mortality [5].

The risk of developing type 2 diabetes and obesity has been linked with alterations in intestinal microbiome, which plays a major role in several metabolic pathways, energy harvesting and regulation of gut hormone secretion [6, 7]. Exposure to antibiotics profoundly affects the composition and function of the intestinal microbiome and has been associated with a range of adverse immunological and metabolic health outcomes [8]. Previous studies using administrative health records suggest that exposure to antibiotics may be associated with increased risk of developing type 2 diabetes [9–11]. However, subsequent results from a large, nested case-control study suggest that these associations from studies relying only on administrative health data might be confounded, as no association between use of systemic antibiotics and type 2 diabetes was evident after adjustment for clinical and lifestyle factors [12]. Antibiotic exposure has also been associated with subsequently increased body mass index (BMI) in early life, but this relationship has not been widely studied in adults [13, 14]. We have previously reported that infection-related hospitalization in childhood, which usually results in treatment with broad-spectrum antibiotics, is associated with an adverse cardiometabolic profile in adulthood [15].

In this study, our aim was to investigate the relationship between prior exposure to systemic antibiotics and the risk of incident type 2 diabetes and overweight/obesity. Using data from the prospective Cardiovascular Risk in Young Finns Study (YFS) of 2209 participants with comprehensive data including potentially confounding early life risk factors, we examined whether exposure to antibiotics between 1993 and 2001 was associated with subsequent incident type 2 diabetes and overweight/obesity. Cross-sectional and prospective data from four National FINRISK studies conducted in 1997, 2002, 2007 and 2012 (*N* = 24,674 in total) were used as replication cohorts.

## Materials and methods

### Study design and participants

YFS was launched in 1980 when 3596 participants 3–18 years of age were examined. Subjects were randomly selected from the national register from different parts of Finland to produce a representative sample of Finnish children. Thereafter, follow-up studies have been conducted regularly and a detailed description of YFS has been published previously [16]. The three latest follow-up surveys were completed in 2001 (from now on referred as “baseline study” for clarity), 2007 and 2011 by 2283, 2204 and 2063 participants, respectively. The study has been approved by the Joint Commission on Ethics of the Turku University and the Turku University Central Hospital and has been conducted according to the guidelines of the Declaration of Helsinki. An informed written consent was obtained from the parents when participants were still under-aged and after reaching adulthood, from the participants themselves.

The FINRISK population surveys have been performed every 5 years since 1972 to investigate trends in cardiovascular disease risk factors in the Finnish population. The FINRISK 1997, 2002, 2007, and 2012 studies were based on stratified randomly selected samples of the population aged 25–74 years, as previously described [17]. Altogether, 27,876 individuals participated in FINRISK studies between 1997 and 2012. An ethical approval was obtained according to the Finnish legislation and common ethical requirements at the time of each survey: confidentiality, anonymity and data protection have been assured. A written informed consent has been obtained from each participant. Surveys have obtained permissions from the ethics committee which has varied over time. For the latest two surveys, in 2007 and 2012, the approval was obtained from the Coordinating Ethics Committee for the Helsinki and Uusimaa Hospital District.

The total study cohort comprised 26,883 participants. In the YFS there were 2209 participants (mean age 31.6 years at baseline (2001), 55.3% female, 110 cases of incident diabetes, 57.0% overweight or obese); and in FINRISK there were 24,674 participants (mean age 49.8 years at baseline (1997, 2002, 2007 or 2012), 53.0% female, 1, 866 cases of incident diabetes, 59.4% overweight or obese). Schematic representation of the study design is shown in Supplemental Fig. 1.

### Antimicrobial exposure

Antimicrobials for systemic use are only available by physician’s prescription and are dispensed in registered pharmacies in Finland. Purchased medications are reimbursed and registered in the Drug Prescription Register maintained by the Social Insurance Institution of Finland [18]. Information on all systemic antimicrobials was extracted from the Drug Prescription Register for the following Anatomical Therapeutic Chemical Classification -codes: J01* (antibiotics for systemic use), J02* (antifungals for systemic use), and J05* (antivirals for systemic use). For YFS, data were available for antimicrobials purchased between 1993 and 2001, prior to the baseline study conducted in 2001 [19]. For FINRISK, data for purchased antibiotics between 1993 and baseline of each survey were available.

### Type 2 diabetes

In YFS, participants were classified as having type 2 diabetes if at any of the clinical examinations (2001, 2007, or 2011) their fasting glucose was ≥7 mmol/L or if they reported having been given a type 2 diabetes diagnosis by a physician [20]. Moreover, individuals whose GHb was ≥6.5% (48 mmol/mol) at the 2011 follow-up or those who reported taking glucose-lowering medication at the 2007 or 2011 follow-up were classified as having type 2 diabetes. We also extracted data on type 2 diabetes diagnoses from the Finnish Hospital Discharge Register, maintained by the National Institute for Health and Welfare [21]. Finally, data on reimbursed medications for type 2 diabetes, indicating a confirmed diagnosis, were acquired from the National Social Insurance Institution’s Drug Reimbursement Registry. Participants with prevalent diabetes at baseline (2001) were excluded from the analyses.

In FINRISK, the diagnoses of prevalent and incident type 2 diabetes were based on the Finnish Hospital Discharge, Drug Reimbursement, and Causes of Death Registers, as previously described [22]. FINRISK participants with prevalent diabetes were excluded from the analyses.

### Weight and obesity/overweight

Weight was measured to the nearest 0.1 kg and height to the nearest centimeter. BMI was calculated as weight (kg) divided by the square of height (m^2^). Overweight/obesity was defined as BMI > 25 kg/m^2^.

### Covariate data

In YFS, information on early life parental smoking, physical activity and parental socioeconomic status were obtained from questionnaires. At 1980 and 1983, parents were asked whether they had ever smoked daily for at least 1 year as an indication of regular smoking. Families were classified into three categories: “no regular parental smoking”; “1 parent has smoked daily for at least 1 year” and “both parents have smoked daily for at least 1 year”. Data collected in study years 1980, 1983, and 1986 were used to estimate physical activity. At ages 3 and 6 years, a physical activity index was calculated from the parents’ ratings of the amount and vigorousness of their child’s play time and the child’s general level of activity [23]. At ages 9 to 24 years, data on frequency and intensity of physical activity during leisure time were acquired with a self-administered questionnaire [24]. The values for physical activity indices were standardized and the average value was used as a measure of physical activity. The annual income of the family income reported by parents of the participants in 1980 was used to represent socioeconomic status. The questionnaire included 8 income categories, which were later converted into present-day values ($). Standard methods were used for measuring blood pressure and fasting serum insulin levels as previously described in detail [16]. Serial data on insulin levels, systolic blood pressure, and BMI collected in clinical examinations in 1980, 1983, and 1986 was used to calculate the area under the curve for these variables between ages 6 and 24 years representing a long-term exposure to each measured attribute [25]. Questionnaires using self-reports were completed to collect data on dietary habits during the past month and these questionnaires included information on consumption of fruits, vegetables, meat, meat products (e.g. sausages and cold cuts), buns, beverages, sweets, and ice cream. Participants selected one of six response categories: (1) not at all or hardly ever; (2) once or twice a month; (3) once a week; (4) a few times a week; (5) nearly every day; and (6) every day. Consumption of milk was assessed as glasses per day.

In FINRISK, information on smoking, physical activity and socioeconomic status were obtained from questionnaires. Smoking was defined as self-reported daily smoking. Leisure-time physical activity was assessed using a four-category question ranging from 1) “In my leisure time I read, watch TV, and work in the household with tasks which do not make me move much and which do not physically exhaust me”; to 4) “In my spare time I regularly exercise several times a week in competitive sports” [26]. Annual household income was used as an indicator of socioeconomic status and questionnaires included 9 income categories in 1997, 2002, 2007, and 10 income categories in 2012. Systolic blood pressure measurements performed by a nurse using a mercury sphygmomanometer were made from the right arm in a sitting position with at least a 5-min rest before the measurement. A mean of two measurements was used. Fasting plasma insulin (*n* = 7520) and glucose (*n* = 7531) levels were measured only in a subsample of individuals who participated in the FINRISK 2002 or 2007 studies.

### Statistical analyses

Mean ± standard deviation, median [interquartile range], and percentages were used, as appropriate, to describe the variables. In YFS, logistic regression models were used to examine association between antimicrobial exposure (from 1993 to 2001) and the incidence of subsequent type 2 diabetes. Likewise, logistic regression models were used to calculate odds ratios (OR) and 95% confidence intervals (CI) to examine the relationship of prior antimicrobial exposure and prevalence of overweight/obesity at the most recent clinical follow-up (2011). Logistic regression models were used as accurate data on the timing of type 2 diabetes diagnoses was not available in YFS. Association between prior antimicrobial exposure and body mass index in 2011 was estimated using linear regression models. In FINRISK, Cox proportional hazards models were used to assess the association between antimicrobial exposure prior to baseline and risk of developing type 2 diabetes (with follow-up ending on December 31, 2016). Logistic and linear regression were used to assess the association of prior antimicrobial exposure with prevalent obesity and body mass index in FINRISK, respectively. All analyses were adjusted for age and sex, and additionally for study cohort in FINRISK. Sex × antimicrobial exposure was studied to investigate if the associations were similar in males and females. Furthermore, analyses were adjusted for potential confounders including childhood family income, parental smoking and early life (age 6 to 24 years) BMI, insulin, systolic blood pressure and physical activity in YFS. Moreover, to examine if the observed risk increase for type 2 diabetes was mediated by excess body weight, analyses were additionally adjusted for BMI measured at the most recent clinical follow-up in 2011 in YFS. In FINRISK, analyses were first adjusted for age, sex, study cohort, BMI (only type 2 diabetes analyses), income, smoking, systolic blood pressure, and physical activity. These analyses were additionally adjusted separately in a subgroup of individuals with available data for insulin (*n* = 7520) and glucose levels (*n* = 7531). Prior exposure to antibiotics was quantified by dividing YFS study population according to exposure quartiles, and following groups were used: 0–1 prior antibiotic courses (29.0% of the participants), 2–3 prior antibiotic courses (24.4% of the participants), 4–6 prior antibiotic courses (23.1% of the participants), and over 7 prior antibiotic courses (23.6% of the participants). In order to compare our findings to previous studies [10, 11] and further examine the relationship between prior antibiotic exposure and type 2 diabetes and overweight/obesity logistic regression models were fitted to compare participants with exposure of > 5 antibiotic courses versus participants with 0–1 antibiotic courses. Incidence of type 2 diabetes per 1000 person-years in these groups was calculated as (cases of incident type 2 diabetes/person-years in the follow-up) *1000. Furthermore, prevalence of overweight/obesity and mean levels of BMI in these groups were calculated. Similar groups were used in FINRISK to replicate the results.

In sensitivity analyses, antibiotic courses within 6 months before the 2001 follow-up were excluded to account for possible reverse causation (i.e. early undiagnosed type 2 diabetes may be associated with increased infections, or symptoms of early type 2 diabetes may be misdiagnosed as an infection, leading to antibiotic treatment). Furthermore, analyses adjusted additionally for dietary habits were conducted. Finally, associations between the three most common antibiotic courses in our study cohort and subsequent type 2 diabetes and overweight/obesity were examined separately in YFS. Participants with exposure of > 5 courses for each antibiotic were compared to participants with exposure of 0–1 courses for any antibiotics.

Differences between participants according to antibiotic exposure quartiles were examined using linear regression for continuous variables, logistic regression for dichotomous categorical variables, and generalized linear models for trichotomous categorical variables. Variables with skewed distribution were square root-transformed before these analyses.

Statistical tests were performed using SAS version 9.4 (SAS institute, Inc., Cary, NC) and R version 3.6.1 with statistical significance inferred at a 2-tailed *P*-value < 0.05.

## Results

### Characteristics of the study population

Characteristics of the study population are shown in Supplemental Tables 1 and 2. To examine whether participants of YFS differed before the exposure period, characteristics in 1980 stratified by antibiotic exposure quartiles are presented in Supplemental Table 3. Participants in the higher exposure quartiles were significantly older and more likely to be female compared to participants with less antibiotic exposure. No other significant differences were observed. The three most common types of antibiotics used were beta-lactam antibacterials, penicillins (ATC-code J01C) (54.2% of the participants in YFS and 29.7% of the participants in FINRISK had used at least one course before the baseline), beta-lactam antibacterials (ATC-code J01D) (53.8% in YFS and 25.7% in FINRISK), and tetracyclines (ATC-code J01A) (46.3% in YFS and 18.7% in FINRISK).

### Antibiotic exposure and type 2 diabetes risk

An increased risk for developing type 2 diabetes was observed in participants with prior antibiotic exposure in YFS (OR 1.043; 95%CI 1.013–1.074) per one antibiotic course. After adjustment for potential confounding risk factors, association remained essentially unchanged (OR 1.034; 95%CI 1.002–1.066). After further adjustment for BMI at the most recent clinical follow-up (2011), the association between antibiotic exposure and type 2 diabetes remained essentially unchanged, though confidence interval widened (OR 1.046; 95%CI 0.996–1.099). The age- and sex-adjusted OR associating antibiotic exposure with type 2 diabetes was 2.29 (95%CI 1.33–3.96) with > 5 versus 0–1 antibiotic courses. The findings were similar after further adjustments (OR 2.07; 95%CI 1.12–3.84). No increased risk for incident type 2 diabetes was observed for prior use of antifungals or antivirals.

The analyses were replicated in FINRISK. Prior exposure to antibiotics was associated with an increased risk for type 2 diabetes (HR 1.022; 95%CI 1.016–1.029) per antibiotic course (Table 1). The results remained similar after adjustments for confounding factors (HR 1.018; 95%CI 1.010–1.026), as they did when additionally adjusted for insulin levels at baseline (HR 1.017; 95%CI 1.006–1.027). Finally, when baseline glucose was added into model as a covariate instead of insulin, the results remained similar (HR 1.022; 95%CI 1.012–1.031). The age-, sex-, and study cohort-adjusted HR associating exposure to antibiotics with type 2 diabetes was 1.73 (95% CI 1.51–1.99) with > 5 versus 0–1 courses. After further adjustments, the HR for incident type 2 diabetes (HR 1.51; 95%CI 1.31–1.73) remained essentially unchanged.

**Table 1:** The odds ratios/hazard ratios (95% CIs) of prior antibiotic exposure (from 1993 to baseline) for subsequent incident type 2 diabetes and obesity/overweight prevalence

		Type 2 Diabetes						Overweight/obesity (BMI > 25)					
		Age- and sex-adjusted^a^			Adjusted with risk factors ^b^			Age- and sex-adjusted^a^			Adjusted with risk factors ^b^		
		OR / HR ^c^	95% CI		OR / HR^c^	95% CI		OR	95% CI		OR	95% CI	
Antibiotics	YFS	1.043	1.013	1.074	1.034	1.002	1.066	1.043	1.019	1.068	1.040	1.009	1.072
	FINRISK	1.022	1.016	1.029	1.018	1.010	1.027	1.023	1.018	1.029	1.027	1.021	1.032
Antifungals	YFS	1.044	0.963	1.133	1.048	0.960	1.144	1.028	0.963	1.097	1.003	0.921	1.091
Antivirals	YFS	1.174	0.958	1.439	1.180	0.958	1.453	1.000	0.847	1.181	1.003	0.921	1.091

Overweight/obesity status defined at baseline (1997, 2002, 2007 or 2012) in FINRISK and at the most recent clinical follow-up (2011) in YFS. OR / HR (95% CI) per one prescribed course of systemic antimicrobial agents

*CI* Confidence interval, *OR* Odds ratio, *HR* Hazard ratio, *YFS* The Cardiovascular Risk in Young Finns Study, *FINRISK* The national FINRISK study

^a^FINRISK analyses adjusted additionally for study cohort

^b^Adjusted for 1) age, sex, childhood family income, parental smoking and early life (age 6 to 24 years) body mass index, insulin, systolic blood pressure and physical activity in YFS. 2) age, sex, study cohort, income, smoking, systolic blood pressure, physical activity, body mass index (not included in the overweight/obesity model) at baseline in FINRISK

^c^Logistic regression (odds ratios) was used in the YFS analyses and Cox’s proportional-hazards model in FINRISK analyses (hazard ratios)

**Fig. 1:**
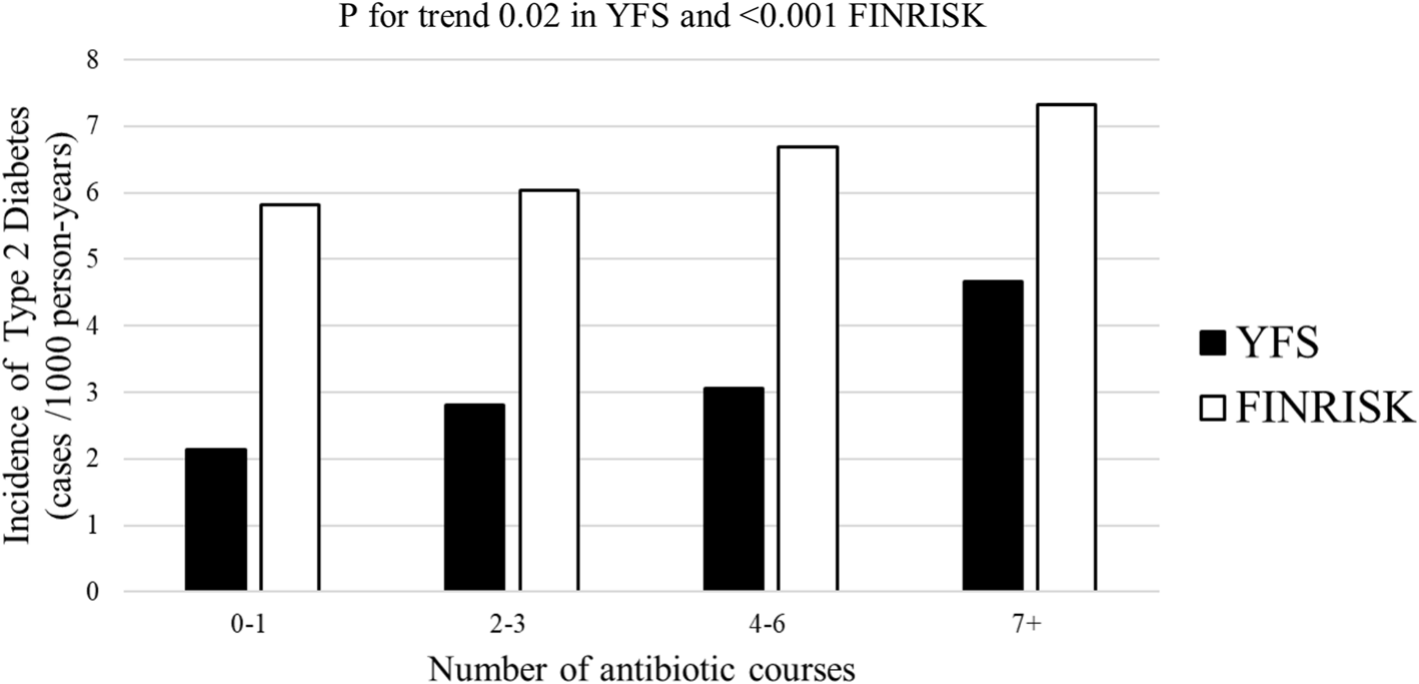
Incidence of Type 2 diabetes from baseline to 2017 according to prior antibiotic medication exposure quartiles (data from 1993 to baseline) in the Young Finns Study and the National Finrisk Study. P for trend was calculated 1) using a logistic regression model adjusted for age, sex, childhood family income, parental smoking and early life (age 6 to 24 years) body mass index, insulin, systolic blood pressure, and physical activity in YFS and 2) using a Cox regression model adjusted for age, sex, study cohort, income, smoking, systolic blood pressure, physical activity in FINRISK

As shown in Fig. 1, the incidence rate for type 2 diabetes increased in a dose-response manner with quartiles of antibiotic exposure in both YFS (P for trend 0.02) and FINRISK (P for trend < 0.001). No interaction between prior antibiotic exposure and sex for incident type 2 diabetes was observed (*P* > 0.05 in YFS and FINRISK).

### Antibiotic exposure and overweight/obesity risk

An increased risk for prevalent overweight/obesity in 2011 was observed in participants with prior exposure to antibiotics (OR 1.043; 95%CI 1.019–1.068) per antibiotic course in YFS (Table 1). After adjustments, the result remained similar (OR 1.040; 95%CI 1.009–1.072). Prior exposure to > 5 antibiotic courses was associated with increased risk for overweight/obesity (age- and sex-adjusted OR 1.57; 95%CI 1.18–2.08) compared to participants who had been exposed to 0–1 prior antibiotic courses in YFS. Further adjustment for confounding factors did not essentially change the results (OR 1.63; 95%CI 1.14–2.34). No increased risk for prevalent overweight/obesity was observed for prior use of antifungals or antivirals.

When analyses were replicated in FINRISK, prior exposure to antibiotics was associated with increased risk for overweight/obesity at baseline (OR 1.023; 95%CI 1.018–1.029) per antibiotic course (Table 1). After adjustment for confounding factors results remained essentially unchanged (OR 1.027; 95%CI 1.021–1.032). Moreover, results remained similar after additionally adjusting for insulin levels at baseline (OR 1.021; 95% CI 1.010–1.032). The age-, sex-, and study cohort-adjusted OR associating exposure to antibiotics with obesity/overweight was 1.49 (95%CI 1.38–1.61) with > 5 versus 0–1 antibiotic course in FINRISK. After further adjustments for potential confounders, the OR remained essentially similar (OR 1.58; 95%CI 1.45–1.71).

As shown in Table 2, prior exposure to antibiotics was directly associated with BMI in 2011 and estimates were β 0.100 ± 0.028 (*p*-value 0.0004) per antibiotic course in YFS. After adjustment for potential confounding risk factors, results remained essentially unchanged (β 0.058 ± 0.023, *p*-value 0.02). No association between exposure to antifungals or antivirals and BMI was observed.

**Table 2:** The associations between prior antibiotic exposure (from 1993 to baseline) with body mass index

		Age- and sex-adjusted^a^			Adjusted for risk factors ^b^		
		β	±SE	*P*-value	β	±SE	*P*-value
Antibiotics	YFS	0.100	0.028	0.0004	0.058	0.023	0.01
	FINRISK	0.063	0.005	< 0.0001	0.068	0.005	< 0.0001
Antifungals	YFS	0.055	0.079	0.49	−0.031	0.067	0.64
Antivirals	YFS	0.059	0.203	0.77	0.145	0.157	0.36

Beta-values are per one prescribed course of systemic antimicrobial agents. Body mass index measured at baseline (1997, 2002, 2007, 2012) in FINRISK and at the most recent clinical follow-up (2011) in YFS

*SE* Standard error, *YFS* The Cardiovascular Risk in Young Finns Study, *FINRISK* The national FINRISK study

^a^FINRISK analyses adjusted additionally for study cohort

^b^Adjusted for 1) age, sex, childhood family income, parental smoking and early life (age 6 to 24 years) insulin, systolic blood pressure, body mass index and physical activity in YFS. 2) age, sex, study cohort, income, smoking, systolic blood pressure, physical activity in FINRISK

In FINRISK, association between prior antibiotic exposure and BMI at baseline was observed (β 0.063 ± 0.005, *p*-value < 0.0001) per antibiotic course and results remained similar after adjustments for confounding factors (β 0.068 ± 0.005, *p*-value < 0.0001) (Table 2). After further adjustment for insulin levels at baseline, result was essentially unchanged (β 0.051 ± 0.008 *P*-value < 0.0001).

**Fig. 2:**
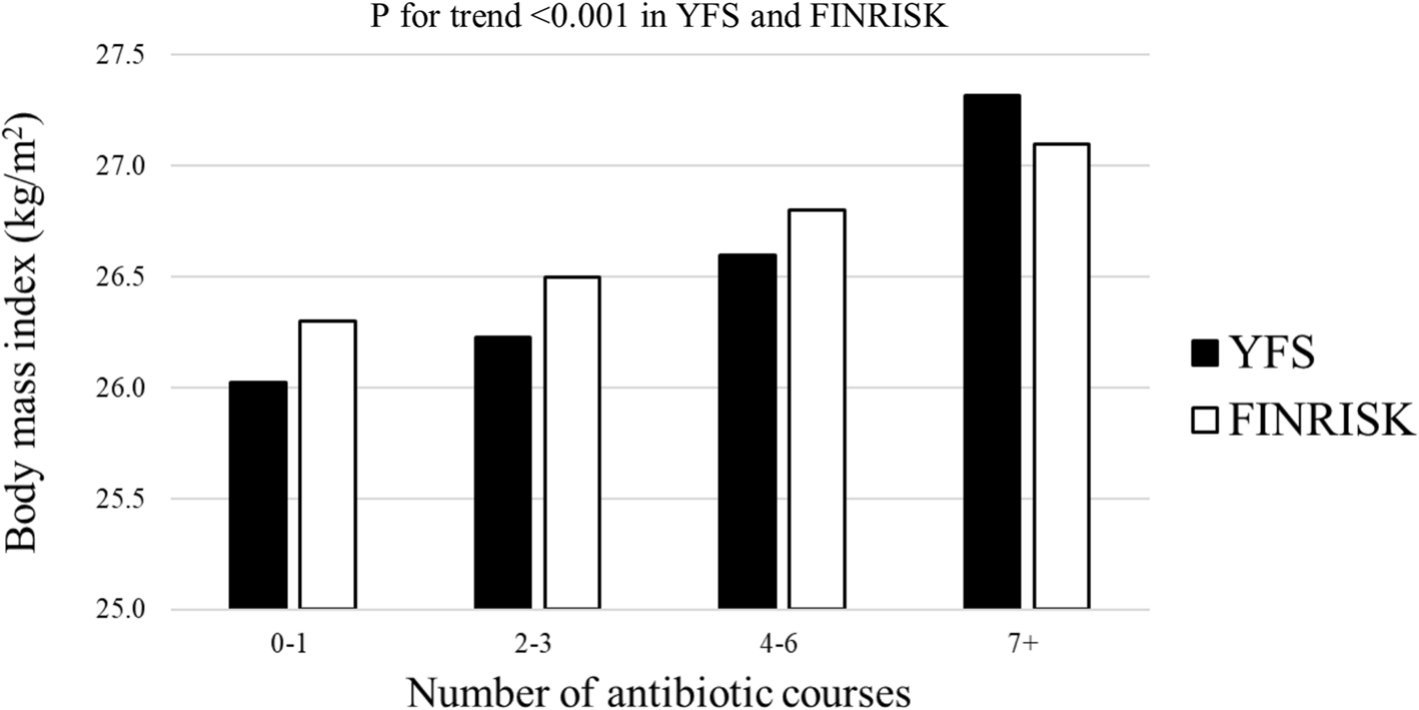
Body mass index according to prior antibiotic medication exposure (quartiles, data from 1993 to baseline) in the Young Finns Study and the National FINRISK Study. P for trend was calculated using a linear regression model adjusted for 1) age, sex, childhood family income, parental smoking and early life (age 6 to 24 years) body mass index, insulin, systolic blood pressure, and physical activity in YFS and 2) age, sex, study cohort, income, smoking, systolic blood pressure, physical activity in FINRISK. Body mass index measured at baseline (1997, 2002, 2007 or 2012) in FINRISK and at the most recent follow-up (2011) in YFS

As shown in Fig. 2 and Supplemental Fig. 2, mean BMI of participants (P for trend < 0.0001 in YFS and 0.004 in FINRISK) and prevalence of overweight/obesity (P for trend 0.0001 in YFS and 0.02 in FINRISK) increased in a dose-response manner with the quartiles of antibiotic exposure. No interactions between sex and prior antibiotic exposure for BMI (*P*-value > 0.05 for all) were observed in YFS and FINRISK.

### Sensitivity analyses

Results remained essentially similar when antibiotic courses purchased within 6 months before the 2001 follow-up were excluded in YFS. The OR for incident type 2 diabetes among those exposed to antibiotics was 1.044 (95%CI 1.013–1.077) per antibiotic course and remained essentially unchanged after adjusting for confounding factors (OR 1.035; 95%CI 1.001–1.069). Furthermore, analyses were additionally adjusted for dietary habits (consumption of fruits, vegetables, meat, meat products (e.g. sausages and cold cuts), buns, beverages, sweets, and ice cream) and the results remained similar, the OR for type 2 diabetes among those exposed to antibiotics was 1.034 (95%CI 1.001–1.069) per antibiotic course and the OR for overweight/obesity was 1.034 (95%CI 1.002–1.067). Finally, prior exposure to > 5 courses of each antibiotic was associated with increased risk for type 2 diabetes (OR 2.86; 95%CI 0.74–11.05 for beta-lactam antibacterials, penicillins, OR 2.94; 95%CI 0.98–8.80 for beta-lactam antibacterials, and OR 1.55; 95%CI 0.32–7.46 for tetracyclines) and overweight/obesity (OR 1.47; 95%CI 0.58–3.73 for beta-lactam antibacterials, penicillins, OR 2.55; 95%CI 1.04–6.27 for beta-lactam antibacterials, and OR 1.37; 95%CI 0.57–3.28 for tetracyclines) after adjustments for confounding factors compared to participants who had been exposed to 0–1 any prior antibiotic courses in YFS.

## Discussion

The results of this study show that prior exposure to systemic antibiotics was associated with increased risk of incident type 2 diabetes in more than 25,000 participants. Furthermore, prior exposure to antibiotics was also associated with overweight/obesity. The observed risks persisted even after adjustment for numerous potential confounding factors, including BMI at baseline. The relationship between antibiotic exposure and type 2 diabetes was not attenuated even after adjusting for BMI from the most recent clinical follow-up. The increasing risk for incident type 2 diabetes and overweight/obesity was cumulative, as participants in quartiles with greater exposure to antibiotics had higher incidence of type 2 diabetes and higher BMI compared to their counterparts with less prior antibiotic exposure.

Four previous studies have examined the relationship between antibiotic exposure and the subsequent incidence of type 2 diabetes [9–12]. First, results from a Danish population-based case-control study, based on national medical records, showed that use of antibiotics was associated with an approximately 53% increase in risk for developing type 2 diabetes in adults receiving ≥5 antibiotic courses compared to those who received 0–1 antibiotic courses [10]. In addition, this study showed that the observed risk for incident type 2 diabetes increased approximately linearly with the antibiotic exposure. Second, data from a similar British study using national medical records, showed a higher risk for incident type 2 diabetes among participants who were exposed to more than one course of penicillin, cephalosporins, macrolides and quinolones [11]. An approximately 23–37% increased risk for type 2 diabetes was observed in adults prescribed > 5 courses of these antibiotics compared to participants who did not receive corresponding antibiotics during the study period. Furthermore, no increase in adjusted risk due to exposure to antiviral or antifungal medications was observed. Third, a retrospective cohort study among U.S. veterans using registry-based data demonstrated that any exposure to systemic antibiotics was associated with approximately 13% increase in risk for type 2 diabetes [9]. Additionally, increased risk for type 2 diabetes was also observed with cumulative total antibiotic prescriptions. Fourth, Alberta’s Tomorrow Project, a nested case-control study from Canada, found that systemic use of antibiotics was not associated with the risk of developing type 2 diabetes after adjustment for clinical and lifestyle data [12]. The authors speculated that previously reported associations observed using only administrative health databases might have been limited in controlling for certain important confounders.

In the present study, we observed that prior exposure to antibiotics was associated with increased risk of incident type 2 diabetes, and this was evident after extensive adjustments for potential confounding risk factors. Furthermore, we observed a dose-response in the relationship between prior exposure to antibiotics and the subsequent risk of developing type 2 diabetes. Alberta’s Tomorrow Project included ~ 15,000 participants (51% women, mean age 59 years, ~ 90% Caucasian) and covariate data was collected from self-administered questionnaires. In contrast, in YFS and FINRISK, data on covariates was obtained by clinical examinations when appropriate and the study population was significantly younger than the study population of Alberta’s Tomorrow Project.

Exposure to antibiotics has been linked with development of obesity and increased BMI in observational studies, mostly conducted during early life [13, 14, 27, 28]. In 2010, Thuny et al. observed that persistent weight gain occurred in patients treated with high doses of vancomycin and gentamycin for infective endocarditis [14]. In the current study, we observed that prior exposure to antibiotic medication was associated with higher body weight and increased prevalence of overweight/obesity. In addition, the association between prior antibiotic exposure and increased BMI persisted over a 10-year follow-up in YFS. Our results also suggest that the relationship between antibiotic exposure and BMI might be cumulative.

The intestinal microbiome has profound effects on human metabolism. Adverse changes to the microbiome have been implicated in development of type 2 diabetes and obesity via several putative mechanisms, including energy harvesting, generation of metabolites and endocrine regulation [29–31]. Animal and human studies have demonstrated that the microbiome from those with obesity is more efficient at harvesting energy from the diet [7, 32]. Furthermore, short chain fatty acids fermented by the intestinal microbiota from dietary fiber have a role in regulation of insulin sensitivity and energy metabolism [33]. Even short-term courses of oral antibiotics may have a long-term impact on intestinal microbial diversity, composition and function [8, 34]. Antibiotic exposure has recently been associated with a range of non-communicable diseases, including obesity and type 2 diabetes, which is mediated by loss of microbiome diversity [35].

Antibiotics are widely used to treat infections, but it has been estimated that 20–50% of the prescriptions are inappropriate (i.e. no health benefit or suboptimal use of antibiotics, such as unnecessarily broad spectrum, an incorrect dosage or duration) [36, 37]. We observed that one antibiotic course increased the risk for subsequent incident diabetes by 1.8–3.4%. In the United States, annual outpatient prescription rate for antibiotics was 0.9 / per capita in 2012; higher than in Scandinavia but lower than many Southern Europe countries [38]. Moreover, participants who had been treated with > 5 antibiotic courses had a substantially increased risk (adjusted OR 1.51–2.07) for future type 2 diabetes compared to participants who had 0–1 antibiotic course during the study period. When analyses were further adjusted for BMI at the most recent clinical follow-up in 2011, the OR remained essentially similar, though confidence interval widened suggesting that the relationship between antibiotic exposure and type 2 diabetes is not completely mediated by excess body weight.

The findings of the present study support prudent use of antibiotics. However, our observational study design cannot infer causality and further studies are needed. A competing interpretation of our findings would be that participants with subsequent type 2 diabetes are more prone to develop infections in the years prior to the type 2 diabetes diagnosis and thus use more antibiotics. Earlier studies have also indicated that excess body weight is associated with more susceptibility to infections [39, 40]. Nonetheless, greater increases in the infection risk are largely seen in individuals with severe obesity which was uncommon in our study population. Also in support for the first interpretation, no substantial differences were observed in the 1980 characteristics including BMI, insulin and glucose levels when participants of YFS were stratified according to the subsequent antibiotic exposure quartiles Moreover, based on the observational data it is not possible to differentiate whether the relationship observed between antibiotic courses and metabolic outcomes is due to treatment (antibiotics) or infections per se. Studies focusing on the direct impact of antibiotic exposure on human gut microbiome are needed in the future to further investigate this question.

Strengths of this study include the large study population of over 25,000 Caucasian participants with comprehensive information on confounding clinical and lifestyle risk factors. We were able to account for possible reverse causation bias in sensitivity analyses that included only antibiotic prescriptions at least 6 months prior to clinical follow-up in 2001. However, as the study population was racially homogenous, the generalizability of our findings is limited to white European subjects. Our exposure variable was a purchased, systemic antibiotic course, but we did not have data on medication adherence or the exact dosage of antibiotics. Poor compliance is unlikely to introduce systematic bias but may lead to underestimation of the true effect size. This study focused on outpatient pharmacy data of oral prescriptions of antibiotics, and therefore does not capture the total antibiotic exposure from intravenous antibiotics given in inpatient settings. However, most patients receiving intravenous antibiotic therapy in hospitals were probably sequentially discharged on oral antibiotics to outpatient settings and this is unlikely to substantially bias our results. Furthermore, logistic regression models were used in YFS as accurate data on the timing of type 2 diabetes diagnoses was not available for all of the participants. Earlier studies suggest that dichotomising time-to-event outcomes may be adequate for low event probabilities [41]. As the rate of incident type 2 diabetes was low in YFS (5.0%), we believe that using logistic regression is not likely to notably bias our results. Finally, non-participation at follow-up is inevitable in longitudinal studies. However, the study group has been dynamic and thus the present study population was probably representative of the original population [42].

## Conclusion

We found that prior exposure to systemic antibiotics was associated with increased risk for future type 2 diabetes and overweight and obesity. Our findings support judicious use of antibiotics. Future studies are needed to further investigate the relationship between antibiotic exposure and type 2 diabetes and excess body weight as well as to examine the direct impact of antibiotic exposure on human gut microbiome.

## Supplementary Information

**Additional file 1.**

**Additional file 2.**

**Additional file 3.**

## Data Availability

The datasets analyzed in this study are not publicly available for ethical and legal reasons but are available from the Publication Committee of the YFS on reasonable request. For more information on requests related to dataset access, please contact Professor Olli Raitakari, Project Director of the YFS, University of Turku, Finland, olli.raitakari@utu.fi.
