# Supplementary material for: Use of antibiotics and risk of type 2 diabetes, overweight and obesity: the Cardiovascular Risk in Young Finns Study and the national FINRISK study
Source: BMC Endocr Disord. 2022 Nov 18;22:284. doi: 10.1186/s12902-022-01197-y (PMC9673285; doi:10.1186/s12902-022-01197-y)
Supplement: Supplementary file 1 — Additional file 1. [file 12902_2022_1197_MOESM1_ESM.docx]

**Supplemental figure legends.**

**Supplemental Figure 1**. Schematic representation of study design.

**Supplemental Figure 2.** Obesity/overweight (BMI >25 kg/m2) prevalence at baseline according to prior antibiotic medication exposure quartiles (data from 1993 to baseline) in the Young Finns Study and the National FINRISK Study.

P for trend was calculated 1) using a logistic regression model adjusted for age, sex, childhood family income, parental smoking and early life (age 6 to 24 years) body mass index, insulin, systolic blood pressure, and physical activity in YFS and 2) using a linear regression model adjusted for age, sex, study cohort, income, smoking, systolic blood pressure, physical activity in FINRISK.

Baseline = 2001 in YFS; 1997, 2002, 2007 or 2012 in FINRISK. Body mass index measured at baseline (1997, 2002, 2007, 2012) in FINRISK and at the latest clinical follow-up (2011) in YFS.

|  | | | Women (N=1223) | | Men  (N=986) | |
| --- | --- | --- | --- | --- | --- | --- |
| Age (years) | | | 10.7 | 5.0 | 10.7 | 5.0 |
| Body mass index (kg/m^2^) | | | 17.8 | 3.0 | 17.9 | 3.1 |
| Systolic blood pressure (mmHg) | | | 112 | 11 | 114 | 13 |
| Annual family income (% of participants) | | ≤8,700$^*^ | 6.0 | | 4.3 | |
|  |  | >8,700$ to ≤14,500$ | 9.5 | | 9.0 | |
|  |  | >14,500$ to ≤20,300$ | 11.2 | | 12.7 | |
|  |  | >20,300$ to ≤26,000$ | 14.7 | | 15.8 | |
|  |  | >26,000$ to ≤31,800$ | 14.8 | | 13.9 | |
|  |  | >31,800$ to ≤43,400$ | 21.8 | | 22.7 | |
|  |  | >43,400$ to ≤57,900$ | 13.9 | | 13.5 | |
|  |  | >57,900$ | 8.1 | | 8.1 | |
| Parental smoking (% of participants) ^†^ | None | | 29.6 | | 28.5 | |
|  | 1 parent smoking | | 47.9 | | 50.1 | |
|  | Both parents smoking | | 22.5 | | 21.4 | |
| Physical activity  index | | 3-6 years  (range 9-23) | 15.7 | 2.4 | 16.5 | 2.5 |
|  |  | 9-18 years  (range 5-14) | 8.6 | 1.6 | 9.6 | 1.9 |
| Insulin (mU/l) | | | 9.5 | [8.5] | 8.0 | [7.0] |
| Fasting glucose (mmol/mol) ^‡^ | | | 4.56 | 0.68 | 4.62 | 0.81 |
| Prior exposure to antimicrobials from 1993 to baseline (2001) (number of prescribtions) | | Antibiotic | 4 (range 0-80) | [6] | 2 (range 0-32) | [4] |
|  |  | Antifungal | 0 (range 0-36) | [0] | 0 (range 0-14) | [0] |
|  |  | Antiviral | 0 (range 0-9) | [0] | 0 (range 0-15) | [0] |

**Supplemental Table 1.** Characteristics in 1980 and antibiotic exposure from 1993 to baseline (2001) in The Cardiovascular Risk in The Young Finns Study.

Values presented as mean ± standard deviation or median [interquartile range] or proportions (percentages).

^*^Family income in 1980. Values were converted from Finnish Marks into corresponding values in 2018 and presented in USD ($).

^†^ Data collected in 1980 and 1983.

^‡^ Glucose was measured in 1986 in 1,061 women and 876 men.

|  | | | Women (N=13,083) | | Men (N=11,591) | |
| --- | --- | --- | --- | --- | --- | --- |
| Baseline study year (% of participants) | 1997 | | 26.3 | | 26.9 | |
|  | 2002 | | 32 | | 31.5 | |
|  | 2007 | | 21.9 | | 21.7 | |
|  | 2012 | | 19.8 | | 19.9 | |
| Age (years) | | | 47 | 13 | 48 | 13 |
| Body mass index (kg/m2) | | | 26 | 5 | 27 | 4 |
| Systolic blood pressure (mmHg) | | | 131 | 20 | 137 | 18 |
| Annual household income categories | | 1 (lowest) | 7.1 | | 6.7 | |
|  |  | 2 | 14.9 | | 12.8 | |
|  |  | 3 | 18 | | 15.8 | |
|  |  | 4 | 15.7 | | 16.4 | |
|  |  | 5 | 13.2 | | 13.6 | |
|  |  | 6 | 11.5 | | 12.4 | |
|  |  | 7 | 7.5 | | 7.9 | |
|  |  | 8 | 4.7 | | 5.3 | |
|  |  | 9 (highest)† | 7.3 | | 9.1 | |
| Daily smoking (% of participants) | | | 19.1 | | 28.5 | |
| Leisure-time physical activity  index  (% of participants) | | Low physical activity | 21.6 | | 20.9 | |
|  |  | Light exercise at least 4 h/week. | 54.7 | | 50.4 | |
|  |  | Higher intensity exercise at least 3 h/week | 23.1 | | 26.1 | |
|  |  | Competitive sports at least several times a week | 0.6 | | 2.6 | |
| Insulin (mU/l)* | | | 5.9 | [4.2] | 6.3 | [5.0] |
| Glucose (mmol/mol) | | | 5.6 | 0.6 | 6.0 | 0.9 |
| Prior exposure to antibiotics from 1993 to baseline (number of prescribtions) | | | 2 (range 0-86) | [6] | 3 (range 0-129) | [6] |

**Supplemental Table 2.** Characteristics at baseline (1997, 2002, 2007 or 2012) and antibiotic exposure from 1993 to baseline in the National FINRISK Study.

Values presented as mean ± standard deviation or median [interquartile range] or proportions (percentages).

*Plasma insulin (n=7,520) and glucose (n=7,531) levels were measured only in a subsample of individuals who participated in the FINRISK 2002 or 2007 studies.

†Income categories 9 and 10 in 2012 were combined.

|  | | Antibiotic exposure (number of prescriptions) | | | | | |
| --- | --- | --- | --- | --- | --- | --- | --- |
|  |  | 0-1 | 2-3 | 4-7 | 7+ | P for difference^*^ | |
| n | | 642 | 533 | 512 | 522 |  | |
| Sex (female %) | | 39 | 51 | 62 | 73 | <0.0001 | |
| Age (years) | | 10.3±5.1 | 10.6±5.1 | 11.0±4.7 | 11.0±5.0 | 0.003 |  |
| Body mass index (kg/m2) | | 17.7±3.1 | 17.7±2.9 | 18.1±3.2 | 18.0±3.1 | 0.37 |  |
| Systolic blood pressure (mmHg) | | 112±12 | 113±12 | 114±12 | 113±12 | 0.15 |  |
| Annual family income (range 0-8) | | 4.8±2.0 | 4.8±2.0 | 5.1±1.9 | 4.8±1.9 | 0.78 |  |
| Physical activity index | 3-6 years (range 9-23) | 16.4±2.3 | 15.9±2.5 | 16.0±2.5 | 15.8±2.5 | 0.43 |  |
|  | 9-18 years (range 5-14) | 9.1±1.8 | 9.1±2.0 | 9.1±1.8 | 8.9±1.8 | 0.53 |  |
| Insulin (mU/l) | | 9.55±6.34 | 9.67±5.78 | 9.80±5.71 | 10.02±5.82 | 0.14 |  |
| Fasting serum glucose (mmol/mol) ^†^ | | 4.63±0.84 | 4.58±0.67 | 4.57±0.70 | 4.58±0.72 | 0.53 |  |
| Parental smoking (% of participants) ^‡^ | None | 28 | 30 | 30 | 28 | 0.52 | |
|  | 1 parent smoking | 50 | 50 | 48 | 47 |  |  |
|  | Both parents smoking | 22 | 20 | 22 | 24 |  |  |

**Supplemental Table 3.** Characteristics in 1980 stratified by antibiotic exposure (between 1993 and 2001) quartiles in the Cardiovascular Risk in the Young Finns Study.

Values presented as mean ± standard deviation or proportions (percentages).

^*^ Linear regression used for continuous variables, logistic regression for dichotomous categorical variables, and generalized linear models for trichotomous categorical variables. Variables with skewed distribution were square root-transformed before these analyses.

^†^ Fasting glucose was measured in 1986 in 1,061 women and 876 men.

^‡^ Data collected in 1980 and 1983.
