# Supplementary figures and images for: Use of antibiotics and risk of type 2 diabetes, overweight and obesity: the Cardiovascular Risk in Young Finns Study and the national FINRISK study
Source: BMC Endocr Disord. 2022 Nov 18;22:284. doi: 10.1186/s12902-022-01197-y (PMC9673285; doi:10.1186/s12902-022-01197-y)

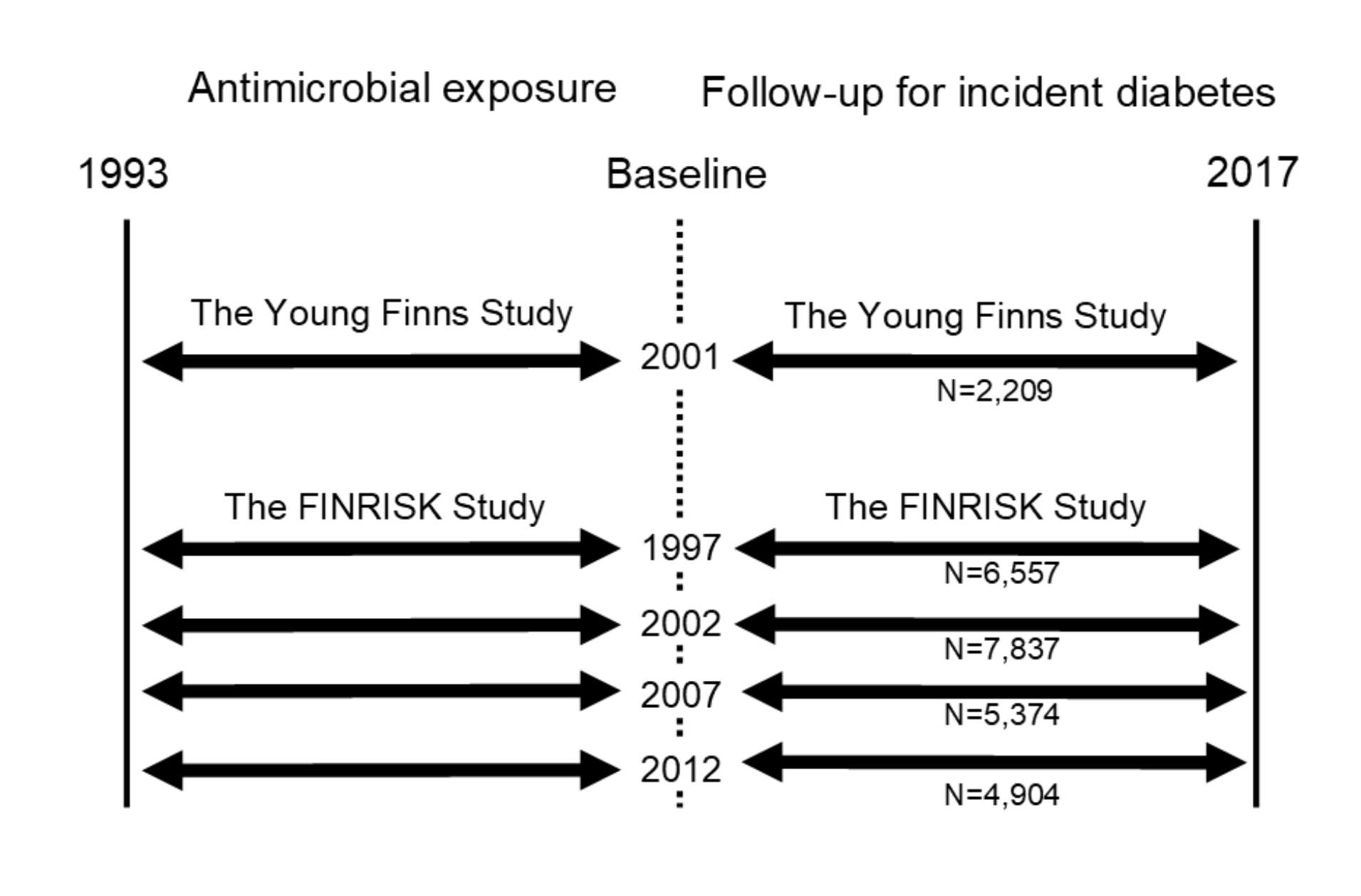

Supplement: Supplementary file 2 — Additional file 2. [file 12902_2022_1197_MOESM2_ESM.tif]

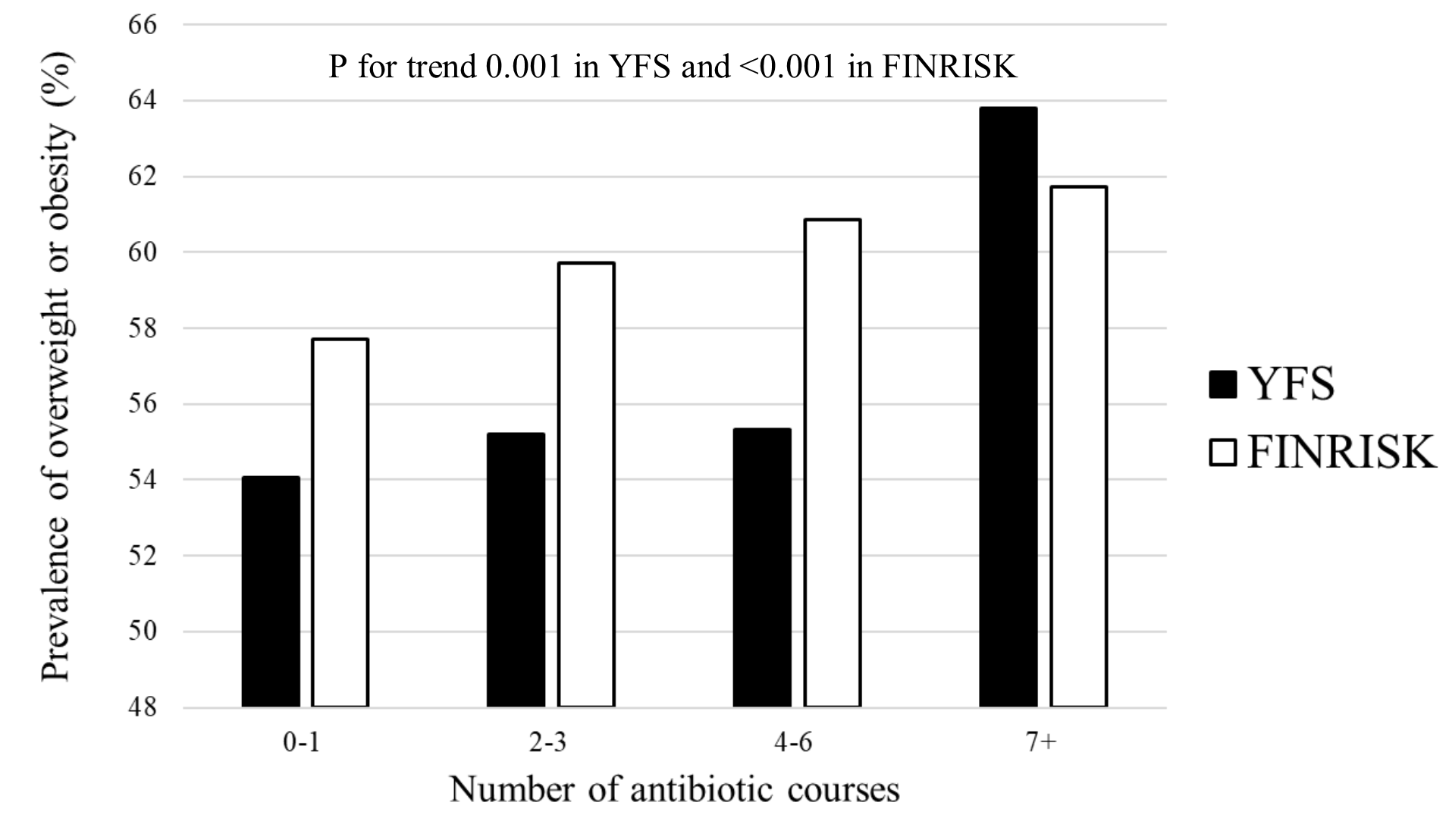

Supplement: Supplementary file 3 — Additional file 3. [file 12902_2022_1197_MOESM3_ESM.tif]
